# Missing the point: re-evaluating the earliest lithic technology in the Middle Orinoco

**DOI:** 10.1098/rsos.180690

**Published:** 2018-06-20

**Authors:** Philip Riris, José Ramón Oliver, Natalia Lozada Mendieta

**Affiliations:** Institute of Archaeology, University College London, 31-34 Gordon Square, London WC1H 0PY, UK

**Keywords:** AMS dating, lithic technology, early Holocene, sediment, preceramic, Orinoco

## Abstract

The Culebra site, located in close proximity to the Atures Rapids, is one of the very few open-air occupations in the entire Orinoco valley that is thought to date to the early Holocene. Following renewed excavations in this location, we characterize the stone technology in unprecedented detail and perform both quantitative and qualitative analyses of the assemblage deposited in the first cultural layers. Additionally, we directly date the sediment forming the depositional context of the assemblage using stratigraphically stable components of soil organic matter. Coupled with our stratigraphic and paedological data, the deposit is, contrary to established estimates, shown to date to the late Holocene, well after the appearance of ceramics in the region. The toolkit identified through the lithic analysis, therefore, does not reflect an Archaic hunter–gatherer adaptation as previously assumed. Our findings are placed in the context of previous research in the Orinoco and lowland South America more broadly. More work is needed to understand the changing role of different stone tool reduction sequences with reference to adaptational strategies and bioclimatic variability.

## 1. Introduction

**Figure 1.**
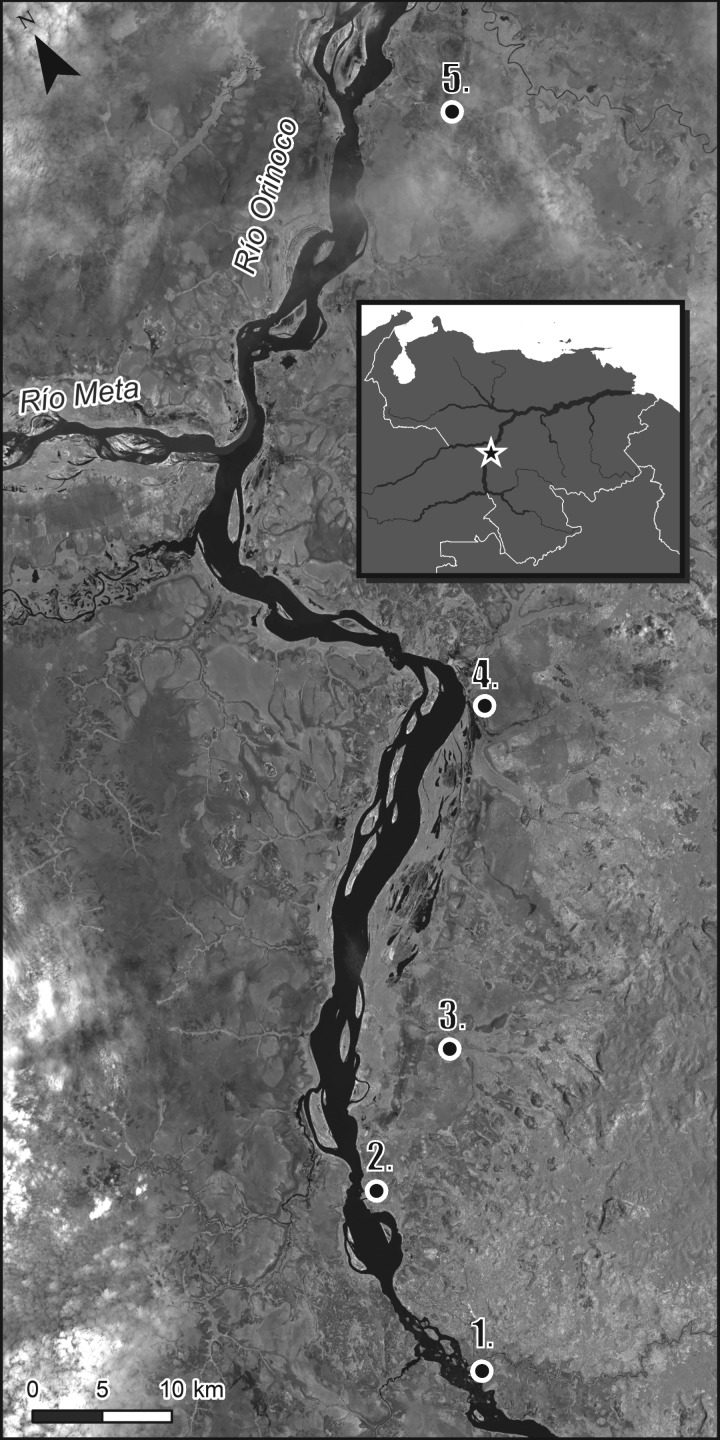
Sites with preceramic deposits along the Middle Orinoco. 1. Culebra, 2. Provincial, 3. Pozo Azul Sur, 4. Lucero, 5. Cerro Gavilán 2. Inset: Location in Venezuela.

Early and Middle Holocene archaeological deposits have been documented in only a handful of sites along the Orinoco River. Here, we provide a re-evaluation of the lithic technology and chronology of the Culebra site, located on a floodplain between the banks of the Orinoco and Cataniapo rivers, in Amazonas State, Venezuela (figure 1). Culebra is the type site for the preceramic Atures I and II phases [1–3], and is one of five other known locations of a similar age in the middle reaches of the Orinoco River. Until recently, the only available radiometric date for deposits estimated to be contemporaneous with Atures I stemmed from the earliest component at the Provincial site, dated to 9020 ± 100 BP (Beta-22638: 8532–7836 cal BC at 2*σ* with IntCal13; [2,4,5]). A recent sequence of dates from the Cerro Gavilán rockshelter [6] extended the earliest human occupation of the Orinoco to 9250 ± 60 BP (Beta-252625; 8621–8308 cal BC) and placed the final preceramic occupation at 3440 ± 40 BP (Beta-252621; 1881–1658 cal BC), capped by ceramic-period occupations. This site also produced abundant faunal and botanical remains, and the rockshelter itself is richly decorated with painted rock art. Nonetheless, the ephemerality of the early and middle Holocene record still presents a challenge to interpreting the lifeways and ecological niches occupied by humans in this environment.

Preceding research has predominantly relied on typological and morphological classification of stone assemblages, particularly projectile points. Additionally, undated deposits such as Atures I and II in the Culebra site are grouped by association with occupations found in the same ‘palaeosol' or B horizon elsewhere along the Orinoco River [2,3,7]. We highlight that: (i) the presence or absence of diagnostic artefacts is the chief criterion for differentiating the hereto undated Atures I and Atures II phases [2], and (ii) the date range of preceramic occupations here is now potentially some six millennia. Adding to this is the predominance of debitage, as opposed to formal tools, reported in these assemblages to date. Long-lived isomorphism in reduction technology between the few known sites implies that either a high degree of consistency was maintained over extreme timespans despite fluctuations in Holocene climate and environment, or that studies to date have not captured some spatially or temporally sensitive aspects of preceramic lithic technology. To contribute towards a resolution of these issues, this paper aims to: (i) furnish a baseline for Atures technological variability, through the characterization of a large sample of stone artefacts obtained in renewed excavations in the Culebra site, and (ii) test the strength of the association between buried soils (‘palaeosols', see [1,2]) and early Holocene occupations along the Orinoco River.

## 2. Material and methods

**Figure 2.**
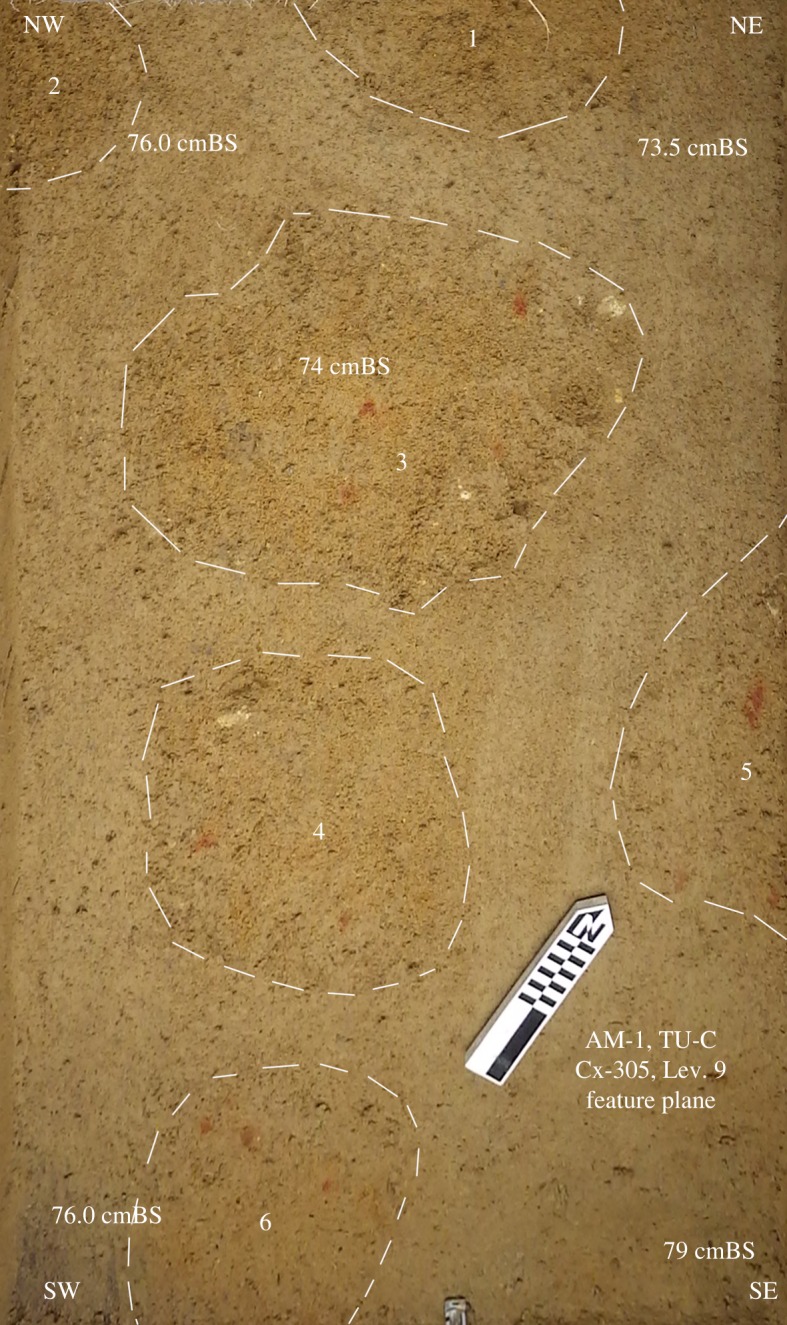
Excavation level 74–79 cm below surface, showing six discrete clusters of lithics associated with red ochre staining and coarser sand.

We conducted excavations in the Culebra site on two occasions: August–September 2015 and January–February 2017. In the first instance, we relocated the original excavations based on information in [1] with the intent to expand the assemblage and obtain material for dating. A 2 × 1 m^2^ trench was placed within 1 m of the trench where the Atures I component was originally reported (Unit N125E50 in [1]) on a north-east/south-west axis, in order to relocate this deposit. Excavations were initially carried out in 10 cm artificial spits using shovels. Besides an ephemeral deposit of heavily eroded ceramic fragments (32 artefacts) between 40 and 50 cm, no artefacts were encountered until the bottom of level 6 (60 cm below the surface onwards). Beyond this point, excavation proceeded with trowels in 5 cm spits. All sediment was screened in a 3 mm mesh, initially dry but subsequently water-screened below 60 cm, as soil moisture increased. All artefacts were collected and recorded. This succeeded in recovering a stone assemblage to a depth of 74–79 cm in a highly leached soil (figure 2), at which point the height of the wet season water table rendered further excavation impossible.

The second round of excavations, during the 2017 dry season, extended the trench by 1 m to the north and 2 m towards the east, resulting in a total area of 9 m^2^ (3 × 3 m) excavated, using the same excavation techniques described above. Excavation was continued in this extended area to a depth of 80 cm. A sudden and marked decrease in the quantity of artefacts was noted below the depth achieved by the 2015 excavations (75–80 cm). A window (15 cm^2^) was opened to a depth of 90 cm to ascertain if the deposits continued, but did not encounter any artefacts below 80 cm. In the absence of preserved macroscopic charcoal, sediment at the base of the excavations was sampled from multiple locations to date the soil organic matter (SOM). We chose to target the insoluble (humin) fraction of SOM for dating with accelerated mass spectroscopy (AMS). As the Culebra site is located on a floodplain close to the water table, dating the humin fraction is less prone to gross errors than using bulk SOM, which contains soluble components that are mobile in the soil profile [8,9]. This characteristic of the humin fraction is advantageous for obtaining average ages for soil horizons in archaeological sites [10]. Nonetheless, we were cautious to avoid sampling patches of ochre-bearing sediment, in the event that these patches are implicated in the vertical movement of soils in the profile. Samples were sieved through a 180 µm sieve and sequentially treated using the ABA (acid–base–acid) method to remove carbonates and alkali-soluble material, before treatment with deionized water and desiccation. The remaining material was employed for AMS dating. Finally, at the conclusion of excavations, data capture was undertaken for reconstruction of the test pit in its finished state using structure-from-motion photogrammetry.

A total of 2144 stone objects were collected from these excavations, of which 65.1% are microdebitage recovered through screening. We performed a quantitative analysis of this assemblage, recording 13 attributes: dimensions (mm), mass (g), raw material (macrocrystalline quartz, microcrystalline (‘milky') quartz and other), scar counts, cortical cover percentage (four-point ordinal scale), the extent and type of retouch received, use-wear presence/absence, platform type (single, crushed, multiple, cortical) and breakage patterns (distal, medial, proximal fragment, or split). Simultaneously, we carried out a qualitative assessment to determine intra-assemblage variability, classify the artefacts according to their place in the reduction sequence, and identify attributes not wholly covered by the metric analysis, such as bipolar reduction [11,12]. Our protocol identified 82 objects (3.8%) as unmodified chunks of stone, predominantly chunks of granular microcrystalline quartz, and a further 232 objects as shatter that cannot be identified as a flake fragment (10.8%). The remaining 416 artefacts are predominantly flakes (18.3%), with a minority of cores and tools (0.51 and 0.46%, respectively) and two hammerstones. The analysis was performed in R [13]. We photographed the artefacts with a Nikon D7100. In figures, we have maintained consistent orientations for each class: flakes, flake tools and cores with proximal ends at the top of the image, and bifacial tools the reverse. Lighting angles and metric scales are consistent between photographs.

## 3. Results

**Figure 3.**
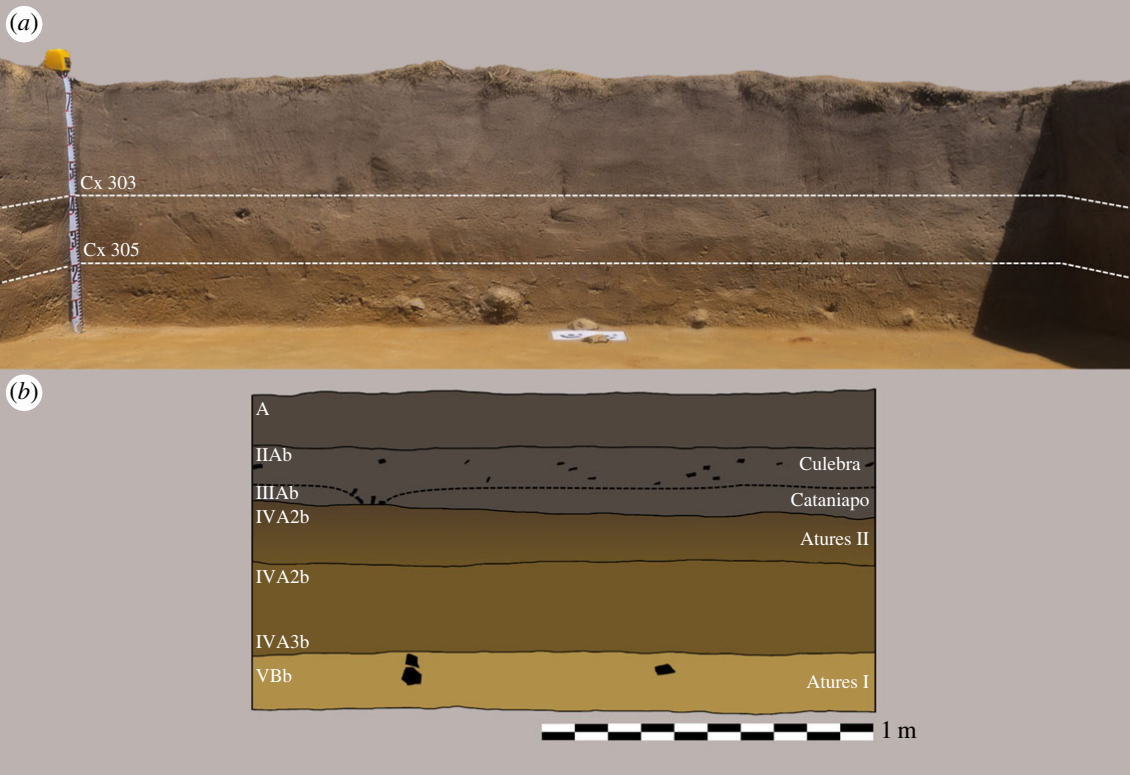
(*a*) 3D render of the Culebra site trench profile, eastern wall, showing the two principal depositional contexts of interest. Main deposit begins at the bottom of Context 303 (60 cm below the surface) and continues uninterrupted until approximately 80 cm (bottom of Context 305). (*b*) Profile of eastern wall redrawn from [1] to scale, with stratigraphic interpretation based on [2]. Colouring based on reported Munsell system codes. Besides very gradual change in colour increasing proportion of sand below 60 cm, we recorded no other stratigraphic or depositional discontinuities.

Excepting the now well-dated site of Cerro Gavilán [6], the preceramic cultural chronology of the Middle Orinoco has been anchored by a single radiometric assay from the ‘Preceramic Component A' layers at the Provincial site. This site is located approximately 15 km downstream from Culebra and argued to be contemporaneous by extrapolating similarities between the soil profiles in these two locations. This practice has, thus far, been the main line of evidence in favour of the antiquity of Atures I at Culebra [2]. Figure 3(*a*) displays the trench profile of our excavations in the Culebra site. We draw attention to the very gradual transition from a dark greyish brown sandy silt to a yellowish brown sandy silt in this profile, which lacks any notable discontinuities in terms of composition, inclusions or compaction until 60–80 cm. At this point, the sandy component of the soil matrix is more coarse-grained with rare, poorly sorted gravelly inclusions that are coeval with sporadic concretions of red iron oxide (probably ochre, figure 2). Contrary to previous findings [1], nowhere did we record loamy sand or sandy loam sediment textures. The change in colour, similarly lacking in marked discontinuities, appears to be the result of heavy leaching and oxidization of the sandy silt soil. Table 1 shows the results of the AMS assays in the Culebra site, taken from the base of the cultural deposits in two locations, slightly separated in depth. These dates, firmly within the late Holocene, are several millennia younger than previously advanced for Atures I (see [2]).

**Table 1.** Results of AMS dating of the soil humin at Culebra. (Calibrated using IntCal13 curve [5].)

laboratory no.	^14^C age (BP)	error	calibrated age (2*σ*)	*δ*^13^C (‰)	depth
Beta-477520	1110	30	AD 879–1013	−14.6	75 cm
Beta-477521	1260	30	AD 669–865	−14.1	79 cm

After the initial appearance of lithic material at 60 cm, we recorded no hiatus in cultural deposits suggestive of two separate periods of occupation (figure 3(*b*)). We observed that all the stone artefacts recorded in these layers *in situ* were similarly orientated flat and concentrated in clusters (figure 2), unlike the heavily eroded and diffuse ceramic fragments from the upper levels of the trench, which suggests the deposit is comparatively less disturbed. Based on the similarity of the material from the top of the deposit and the bottom, our results are presented based on the analysis of the assemblage as a single entity.

**Figure 4.**
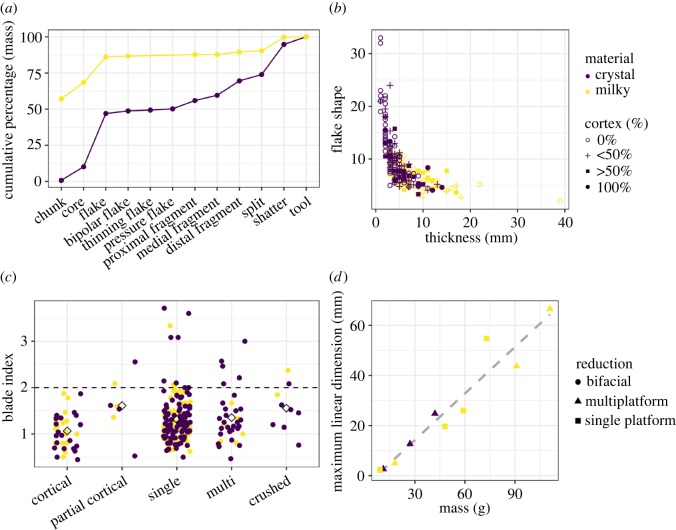
(*a*) Cumulative mass by artefact category and raw material in whole assemblage. (*b*) Distribution of whole flake shape (W + L/T) against thickness, with cortical percentage. (*c*) Blade Index (L/W) by platform type for whole flakes. Flakes with values greater than 2 are considered flakes (Andrefsky [11]). Group mean is displayed as hollow diamonds. (*d*) Linear regression revealing a strong correlation between core maximum dimension and mass.

Figure 4(*a*) shows the cumulative percentage of mass by artefact category. In broad accordance with previous findings [1–3], we characterize the lithic deposits in Culebra as a heavily reduced and fragmented core and flake assemblage, with the majority of flakes (80.1%) presenting no cortical cover whatsoever. In addition to the high incidence of shatter (see above), 37.4% of flakes are fragments and, together, these artefacts make up nearly a quarter of the non-microdebitage assemblage by weight. Although there is a very low incidence of blades overall (figure 4(*c*)), three-quarters of blades are crystal quartz, compared to 65% of all whole flakes. Additionally, a comparison of whole flake relative shape against mass (figure 4(*b*); [14]) suggests the existence of further differences based on raw material properties. Crystal quartz debitage tends towards thin flakes with very low mass on average, although flakes with greater than 50% cortex are generally thicker and heavier. Milky quartz flakes are thicker, shorter and more massive. We note that the few flakes with signs of use-wear (*n* = 20) are overwhelmingly (95%) made from crystal quartz. Only seven flakes, all of them crystal quartz, received retouch in the Culebra assemblage or were shaped into scraping tools. These insights from the metric and observational analyses indicate that there may be important technological differences in the preparation and use of raw materials.

Comparing core mass and maximum linear dimension (as a proxy for maximum potential flake size in the assemblage [11]) provides some insight into the differential attributes of the flake assemblage (figure 4(*d*)). A linear regression of these variables explains a large proportion of the variation in the core assemblage (*R*^2^* = *0.922, *p* < 0.01). Nonetheless, the broader range of size grades of milky quartz cores contrast with the, on average, smaller crystal quartz cores, which are all on rock crystals. Owing to their naturally occurring form as prismatic crystals with six faces, the reduction of crystal quartz cores generally makes use of the long faces as *platforms* rather than removal planes. This fact may be affecting the results of the regression. To address this, we permuted the analysis through each linear dimension for the milky quartz and crystal quartz cores separately. We find that core *length* provides the best fit for crystal quartz (*R*^2^ = 0.948, *p* < 0.01), while *width* provides the best for milky quartz (*R*^2^ = 0.937, *p* < 0.01). Width and thickness were, respectively, the poorest-performing predictors. These findings, supported by the debitage analysis, imply that cobbles of these raw materials possess significantly different reduction sequences and produce debitage with different characteristics.

**Figure 5.**
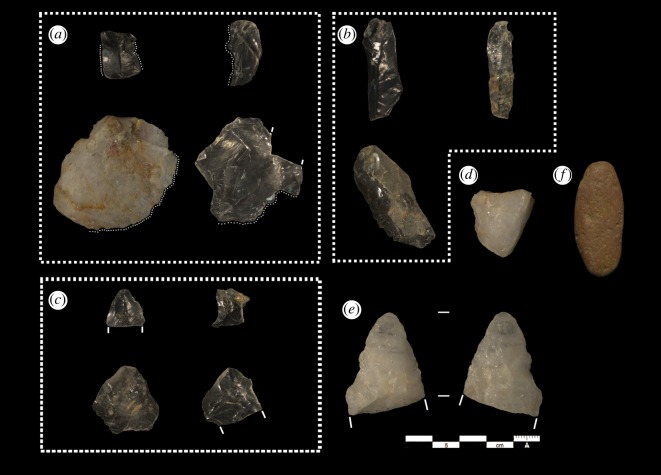
Sample of artefacts from Culebra: (*a*) flakes with use-wear along indicated edges, (*b*) blades on crystal quartz, (*c*) candidates for classification as bifacial thinning flakes, (*d*) milky quartz core, (*e*) broken bifacial preform on milky quartz and (*f*) hammer stone.

The high frequency of microdebitage in the assemblage relative to regular debitage (table 2) as well as the relatively high ratio of broken flakes and shatter to whole flakes indicates that both raw materials are relatively unpredictable. Although ‘rock crystal’ quartz fractures conchoidally, like chert, the granular structure of milky quartz causes irregular fractures. We observe that both tool preforms in the sample (projectile points on milky quartz, figure 5*e*) apparently broke during shaping, and further, that known projectile points from this site [2] might be in the rough-out stages as well. While raw material with irregular fracture mechanics may provide an explanation for the large proportion of shatter in Culebra, we suggest that the microdebitage also consists of the waste products of secondary flaking, shaping and retouch of formal stone tools. Further to this, we have identified crystal quartz flakes with lipped platforms, multi-directional scars and curved profiles, characteristic of biface thinning debitage (figure 5*c*), rather than the prismatic cores encountered in our excavations. These flakes cannot yet be matched with corresponding bifacial artefacts in the same raw material, which are apparently absent from the sample.

**Table 2.** Microdebitage mass analysis by raw material.

material	count	count %	mass (g)	mass %	mass : count
crystal quartz	950	68.0	130	52.9	7.3
milky quartz	440	31.5	110.9	45.2	3.96
other	7	0.5	4.6	1.9	1.52
total	1397		245.5		

## 4. Discussion and conclusion

In this paper, we analysed a lithic assemblage from the Middle Orinoco, Venezuela to provide the first baseline for stone reduction technology in this setting. Our analyses identified several reduction sequences that coexisted at the Culebra site. While there is little variability by excavation level, significant differences can be highlighted between the raw materials in the assemblage. We suggest that this may in part be owing to the fracture mechanics of different quartz species affording different opportunities for reduction, yet which were in parallel usage. To this end, although core and flake technology dominates, our excavation method also succeeded in documenting a large quantity of microdebitage. While it is likely that some proportion of this material is simply shatter, it can be proposed that it provides indirect evidence for bifacial stone tool production at Culebra, a suggestion which is bolstered by the identification of candidates for bifacial thinning flakes among the debitage. Together with the preforms recorded here [2], these elements of the assemblage are strongly suggestive of activities geared towards projectile point preparation, which are missing from the excavated assemblage probably owing to removal and use elsewhere [15], possibly for hunting or spearfishing in the Orinoco River itself.

Nonetheless, our results are unable to confirm the depositional and chronological sequence advanced for the Culebra site previously [1,2]. We refer specifically to: (i) the existence of buried A-horizons of sandy loam overlaying a B-horizon that together form multiple well-defined ‘palaeosols’ and (ii) a 40 cm hiatus between the top and bottom of the Atures I and II cultural deposit. In the first instance, a very gradual change in soil colour is the only significant difference in the profile between the surface until 60 cm below the surface. At this point, the sandy component of the soil becomes coarser and concretions appear. This heavily leached zone is also where the majority of the artefacts were recorded. We stop short of defining the context as a palaeosol or buried surface. The classification of soils as such is a contentious area in palaeopaedology [16,17], and a change in texture in a single soil layer is not sufficient for defining a distinct stratum or horizon in unambiguous terms [18]. Moreover, directly dating this sediment enables us to reject the prior estimate for Atures I and II between 7000 and 4000 BC [2]. Two separate assays on the humin fraction place the likely date of deposition of the Culebra lithic assemblage in the latter half of the first millennium AD. These dates are stratigraphically aligned and do not overlap at 2*σ* calibration ( table 1). We remain open to the possibility that the coarse sand layer may be the remains of an ancient surface. This interpretation is tempting in light of the largest concentration of artefacts occurring on a plane, but cannot be proved definitively. Building chronologies based on cross-referencing ‘palaeosols’ from different sites, which have themselves been defined on the basis of the presence or absence of cultural material [1] remains problematic. Our results suggest that this may overstate the degree of correlation between soil profiles in widely separated riverine sites, a caution that is broadly applicable to the tropical lowlands of South America.

This reassessment, together with the findings of the technological analysis, implies a lack of strong evidence to distinguish Atures I and II phases in the Middle Orinoco. The lithic assemblage discussed in this paper was probably deposited, at a minimum, a millennium after the appearance of ceramics in the Orinoco valley [19,20]. This component of Culebra is, therefore, better described as aceramic rather than preceramic *sensu stricto*. Indeed, contemporary ceramic deposits are known in Culebra itself, the earliest dating to 1450 ± 90 BP (Beta-22640: 407–766 cal AD at 2*σ* [1]). We tentatively interpret the dates and the deposit of lithic material analysed here as the result of relatively time-constrained and specialized activity in Culebra. Going further, and noting the relatively small proportion of the assemblage that indicates bifacial tool manufacture, it is possible that this activity included fishing in the Orinoco and Cataniapo rivers. Other artefacts, such as scraper flakes, might relate to the preparation of catch. Studying patterns of microwear on a larger sample of artefacts from more sites would be able to test if there are other such pre-Columbian fishing stations along the Middle Orinoco. At present, published descriptions of lithic material from the Pozo Azul Sur and Provincial sites are not sufficiently detailed for a technological comparison with the assemblage in Culebra [1,2,7] to ascertain their functional similarities and differences. At a minimum, they are suggestive of unifacial reduction on similar raw materials. This raises the question of when bifacial projectile point technology was adopted or developed in this region, and how it interfaced with the apparently long-lived and widespread unifacial reduction tradition [6,7].

We conclude by commenting on broader questions facing archaeologists studying early to mid-Holocene (Archaic) adaptations in the moist Neotropics. Following our results and discussion, it is likely that formal stone tools defined solely by morphology, in this case projectile points, may be an unreliable chronomarker in the Orinoco without a better understanding of their technological variability. Given the overall rarity of projectile points in the material record compared to the relative abundance of unifacial flaking, a similar argument has been made in the central Amazon ([21], cf. [22–24]). Early and mid-Holocene preceramic sites with secure dates are, as noted, found at Provincial, and Cerro Gavilán 2, associated primarily with unifacial core and flake technology and seemingly without widespread evidence of bifacial stone tools [2,6,7]. Considering the *longue durée* of Archaic occupations in the Middle Orinoco, a priority should be to identify variability in unifacial reduction sequences across the Archaic/Formative divide. If, as our results imply, bifacial projectile points are not necessarily diagnostic of preceramic hunting adaptations, then putative spatio-temporal connections to Archaic deposits as far away as the Sabana de Bogotá [25] should be re-evaluated. Finally, we note that lithic technological conservatism on millennial timescales would not be unprecedented in the South American lowlands [26,27]. Comparative work, additional radiometric assays, better experimental controls and accompanying geochemical and micromorphological work is required to clarify how the role of lithic technology changed over time and relative to bioclimatic variables in the Orinoco basin. We hope that our technological characterization of this assemblage proves to be the first of many steps.

## Supplementary Material

High-resolution images, data files, and code

## Data accessibility

The code and datasets supporting this article have been uploaded as part of the electronic supplementary material.
